# Acid-sensing ion channel (ASIC) 4 predominantly localizes to an early endosome-related organelle upon heterologous expression

**DOI:** 10.1038/srep18242

**Published:** 2015-12-15

**Authors:** Verena Schwartz, Katharina Friedrich, Georg Polleichtner, Stefan Gründer

**Affiliations:** 1 Institute of Physiology, RWTH Aachen University, Pauwelsstrasse 30, D-52074 Aachen, Germany

## Abstract

Acid-sensing ion channels (ASICs) are voltage-independent proton-gated amiloride sensitive sodium channels, belonging to the DEG/ENaC gene family. Six different ASICs have been identified (ASIC1a, ASIC1b, ASIC2a, ASIC2b, ASIC3, ASIC4) that are activated by a drop in extracellular pH, either as homo- or heteromers. An exception is ASIC4, which is not activated by protons as a homomer and which does not contribute to functional heteromeric ASICs. Insensitivity of ASIC4 to protons and its comparatively low sequence identity to other ASICs (45%) raises the question whether ASIC4 may have different functions than other ASICs. In this study, we therefore investigated the subcellular localization of ASIC4 in heterologous cell lines, which revealed a surprising accumulation of the channel in early endosome-related vacuoles. Moreover, we identified an unique amino-terminal motif as important for forward-trafficking from the ER/Golgi to the early endosome-related compartment. Collectively, our results show that heterologously expressed ASIC4 predominantly resides in an intracellular endosomal compartment.

In mammals, four different genes code for at least six distinct acid-sensing ion channel (ASIC) subunits: ASIC1a^1^ and ASIC2a^2^,^3^ and their splice variants ASIC1b^4^,^5^ and ASIC2b^6^, ASIC3^7^ and ASIC4^8^,^9^. Functional ASICs are homo- or hetero-trimeric assemblies of individual subunits^10^. They are activated by a drop in extracellular pH and desensitize during sustained acidification^11^. ASICs are members of the degenerin/epithelial Na^+^ channel (DEG/ENaC) superfamily and share about 25% sequence identity with ENaC subunits^12^.

In heterologous expression systems, ASIC1a, ASIC1b, ASIC2a, and ASIC3 form functional homomeric channels^1^,^3^,^4^,^5^,^7^, while ASIC2b and ASIC4 do not^6^,^8^,^9^. Whereas ASIC2b contributes to functional heteromeric channels^6^, mammalian ASIC4 does also not contribute to functional heteromeric channels, because it apparently does not change the electrophysiological properties of other ASIC subunits, when co-expressed^8^. Thus, ASIC4 is not a bona fide ASIC. It has, however, been reported that, in heterologous expression systems, ASIC4 down regulates the expression of ASIC1a and ASIC3^13^.

There is compelling evidence that ASIC1a, ASIC2a, ASIC2b and ASIC3 contribute to functional ASICs in the plasma membrane of neurons^14^,^15^,^16^,^17^,^18^,^19^,^20^,^21^,^22^. ASIC1b-containing ASICs have not been unequivocally identified in neurons, but the presence of ASIC1b in the plasma membrane of a subpopulation of sensory neurons is likely^4^,^5^. In contrast to all other ASICs, function and location of the ASIC4 protein are unknown. ASIC4 has been cloned from neuronal tissue and its mRNA is faintly expressed all over the brain with highest abundance in pituitary gland^8^. Transgenic reporter mice confirmed strong expression of ASIC4 in pituitary gland and revealed restricted expression in other neurons, including a subpopulation of interneurons and cerebellar granule cells. It is possible that in some, but not all, of these cells ASIC4 is co-expressed with ASIC1a and modulates its expression^23^. It has been reported that ASIC4 is present in the plasma membrane of CHO cells, when heterologously expressed^13^. Thus, although current evidence suggests that ASIC4 is present at the plasma membrane, subcellular location and trafficking of ASIC4 are not well understood.

In this study, we investigated the subcellular location of ASIC4, heterologously expressed in COS-7 and HEK293 cells. We consistently found that ASIC4 mainly localizes to vacuoles related to early endosomes. We found that a conserved amino-terminal domain was important for accumulation in early endosome-related vacuoles. Moreover, we identified a carboxyl-terminal di-arginine motif that retained ASIC4 in early endosome-related vacuoles and prevented its passage to late endosomes. In contrast, we could not detect plasma membrane expression of ASIC4. Collectively, our results show that heterologously expressed ASIC4 mainly resides in an intracellular compartment related to early endosomes.

## Results

### ASIC4 accumulates in early endosome-related vacuoles

**Figure 1:**
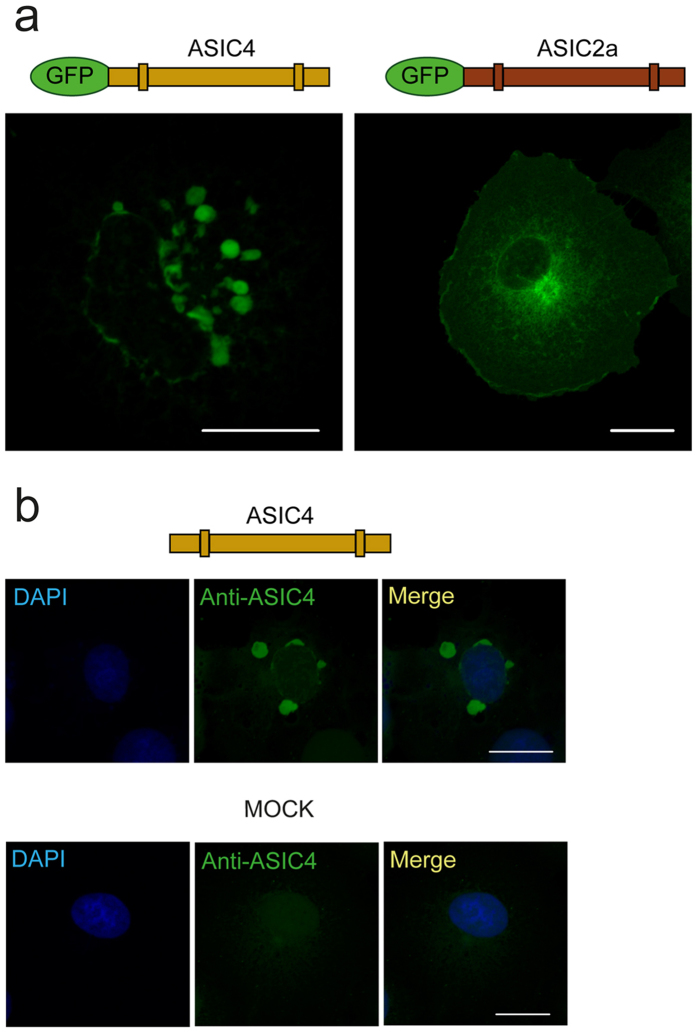
Comparison of subcellular distribution of GFP-ASIC4 and GFP-ASIC2a. (**a**) Left, representative image showing that GFP-ASIC4 mainly accumulated in vacuolar-like structures when expressed in COS-7 cells. Right, in contrast, GFP-ASIC2a had a reticular distribution pattern associated with a slight surface staining. Schemes illustrate the primary structure of ASIC4 (light brown) and ASIC2a (dark brown) with GFP at the amino-terminus (green ellipse) and two transmembrane domains (boxes). Scale bars: 20 μm. (**b**) Staining with an anti-ASIC4 antibody showed similar accumulation of untagged ASIC4 in vacuolar-like structures after expression in COS-7 cells. The secondary antibody was AlexaFluor488-tagged anti-rabbit (green). Untransfected COS-7 cells (MOCK) were not stained by the anti-ASIC4 antibody. Scale bars: 20 μm.

Individual ASIC subunits show a topology with a large extracellular domain, relatively short intracellular amino- and carboxyl-termini and two transmembrane domains^24^. We fused ASIC4 and, for comparison, ASIC2a at their cytoplasmic amino-termini to GFP (GFP-ASIC4 and GFP-ASIC2a, respectively), transiently transfected them into COS-7 cells, and examined their subcellular distribution by confocal laser scanning microscopy. When ASIC1, ASIC2 and ASIC3 are over-expressed in heterologous cells they predominantly localize in the ER^25^,^26^,^27^,^28^. In agreement, GFP-ASIC2a showed a reticular distribution pattern associated with a slight membrane staining, suggesting a predominant location in the ER (Fig. 1a). In stark contrast, GFP-ASIC4 mainly accumulated in large vacuolar-like structures (Fig. 1a). In addition, it usually showed a perinuclear staining, suggesting that GFP-ASIC4 partially localized to the ER. Transfection of GFP-ASIC4 in HEK293 cells revealed a similar accumulation in vacuolar-like structures (see Supplementary Fig. S1 online). We examined cells after different times of transfection (12, 24 and 48 h) to investigate whether GFP-ASIC4 might accumulate in the vacuolar structures after passage through a different cellular compartment. But already after 12 h, GFP-ASIC4 showed the typical vacuolar distribution pattern (see Supplementary Fig. S1 online). ASIC4 fused to GFP at its carboxyl-terminus (ASIC4-GFP) showed an identical distribution (see Supplementary Fig. S1 online), excluding that the position of GFP affected the distribution pattern of ASIC4. Moreover, transfection of COS-7 cells with untagged ASIC4 and staining cells with an anti-ASIC4 antibody revealed an identical accumulation of ASIC4 in large vesicles, excluding that the GFP induced the formation of these vesicles (Fig. 1b). Importantly, the antibody did not stain such vacuoles in untransfected control cells (Fig. 1b). Location in vacuolar structures suggests that over-expression of ASIC4 leads to fusion of vesicles and in accumulation in large endocytic structures. Resembling these findings, it has previously been reported that transient receptor potential mucolipin-1 (TRPML1), a resident protein of lysosomes^29^, when over-expressed in HeLa cells localizes in vacuolar structures containing lysosomal markers^30^, showing that fusion of vesicles is not an indication for mislocalization of an over-expressed channel.

**Figure 2:**
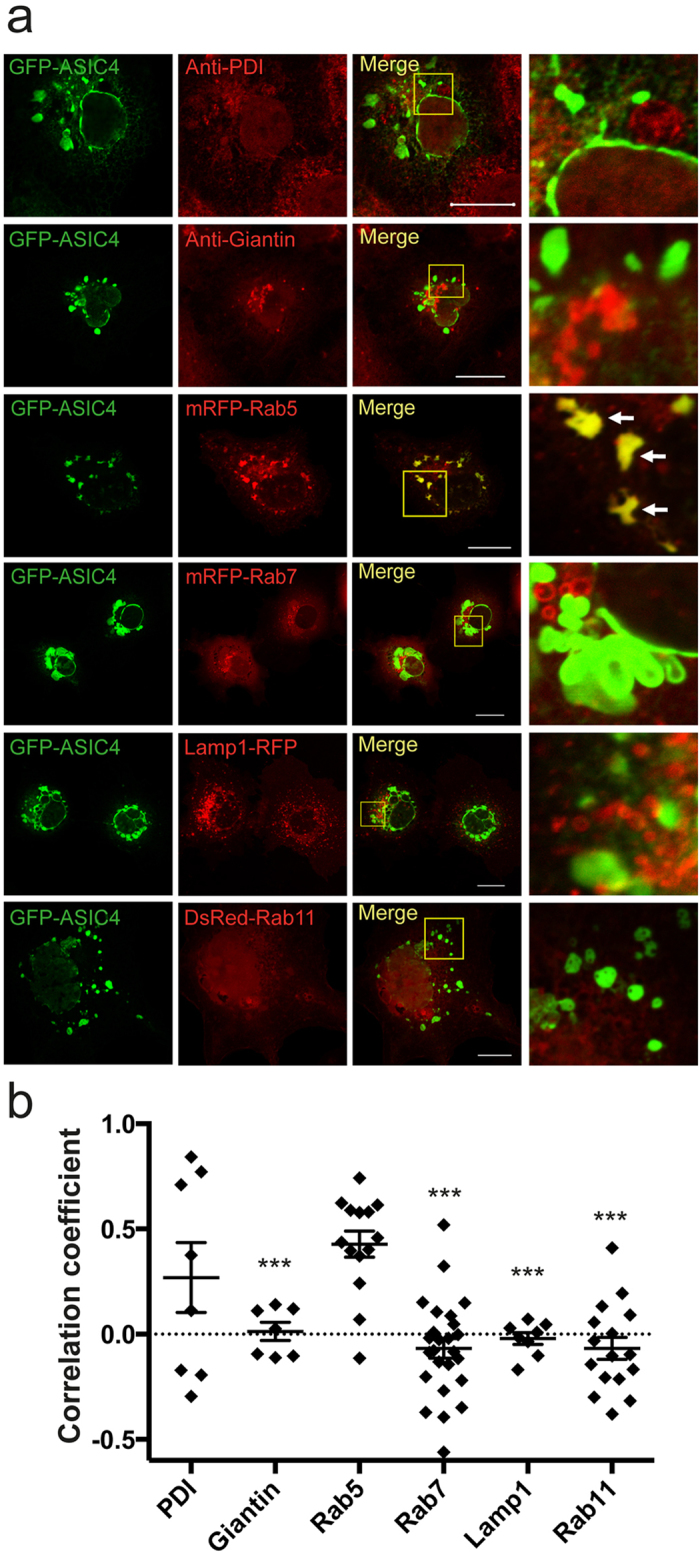
GFP-ASIC4 co-localizes with a marker protein for early endosomes. (**a**) Representative images of GFP-ASIC4 (green) expressed in COS-7 cells. Endoplasmic reticulum was stained with an antibody against the ER-resident protein disulfide isomerase (PDI) and Golgi apparatus with an antibody against the Golgi-resident giantin. To identify endosomes, GFP-ASIC4 was co-expressed with mRFP-Rab5 (early endosomes), mRFP-Rab7 (late endosomes), Lamp1-RFP (lysosomes) and DsRed-Rab11 (recycling endosomes). Images on the right show magnifications of informative regions marked in the “Merge” images. Arrows indicate examples of strong overlap of green and red fluorescence when GFP-ASIC4 was co-expressed with mRFP-Rab5. Scale bars: 20 μm. (**b**) Colocalization was quantified by Pearson´s correlation coefficient (n = 7–25 images). Statistical differences between PCC values of Rab5 and other conditions were analysed by Student´s t-test; ***p < 0.001.

To identify the vacuolar structures, in which ASIC4 accumulated, we used different antibodies and different organelle markers fused to RFP or DsRed. Stainings with an anti-PDI antibody against protein disulfide isomerase of the ER and an anti-giantin antibody against a surface protein of the trans-Golgi-network did not show co-staining with GFP-ASIC4 (Fig. 2a). Similarly, co-expression of GFP-ASIC4 with protein markers for late endosomes (mRFP-Rab7)^31^, lysosomes (Lamp1-RFP)^32^, and recycling endosomes (DsRed-Rab11)^33^ did not reveal co-localization (Fig. 2a). In contrast, co-expression with a protein marker for early endosomes, mRFP-Rab5^31^, showed strong overlap of green and red fluorescence (Fig. 2a), suggesting that in COS-7 cells GFP-ASIC4 accumulated in vacuolar structures that are related to early endosomes. mRFP-Rab5 stained additional vesicles (Fig. 2a), showing that the overlap between Rab5 and ASIC4 staining was not perfect. We quantified co-localization using Pearson´s correlation coefficient (PCC; see Methods). While PCC values for giantin, Rab7, Lamp1 and Rab11 were all close to zero (PCC_Giantin_ = 0.01 ± 0.04, PCC_Rab7_ = −0.07 ± 0.05, PCC_Lamp1_ = −0.02 ± 0.03, PCC_Rab11_ = −0.07 ± 0.05, respectively; n = 7–25), indicating that their location was uncorrelated to ASIC4, PCC for Rab5 was significantly higher (0.43 ± 0.06; n = 14; p < 0.001; Fig. 2b), indicating a partial co-localization with ASIC4. PDI had an intermediate PCC (0.27 ± 0.17; n = 8; Fig. 2b), not significantly different from Rab5, in agreement with the presence of part of the ASIC4 pool in the ER.

**Figure 3:**
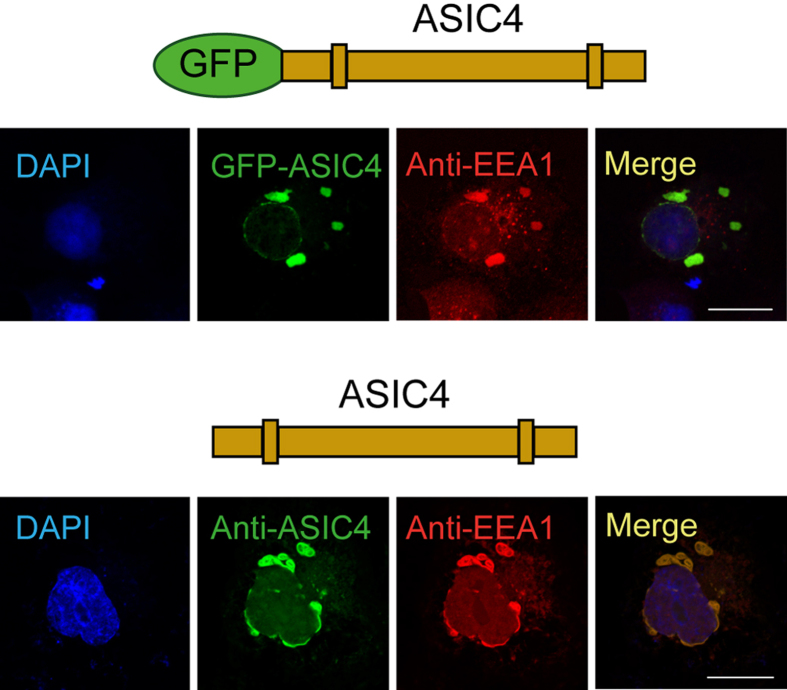
Expression of ASIC4 in early endosomes detected by an anti-EEA1 antibody. Top, in COS-7 cells expressing GFP-ASIC4, staining with an antibody against the early endosomal antigene 1 (EEA1, red) identifies the same organelles as GFP fluorescence. Bottom, co-labelling of ASIC4 and EEA1 in cells expressing untagged ASIC4 (green). Strong overlap of staining for ASIC4 and EEA1 in both cases confirms that over-expressed ASIC4 predominantly localizes to vacuolar structures that are related to early endosomes. Scale bars: 20 μm.

To exclude that over-expression of mRFP-Rab5 resulted in formation of vesicular endosome-like structures or in mislocalization of ASIC4, we transfected GFP-ASIC4 or untagged ASIC4 in COS-7 cells and stained with an antibody against an endogenous marker protein of early endosomes, early endosomal antigen 1 (EEA1). In both cases this revealed a strong overlap of staining for ASIC4 and the marker for early endosomes (Fig. 3), confirming that over-expressed ASIC4 predominantly resides in large vacuolar-like structures that are related to early endosomes.

### Over-expression of ASIC4 traps endocytosed proteins in large early endosome-related vesicles

**Figure 4:**
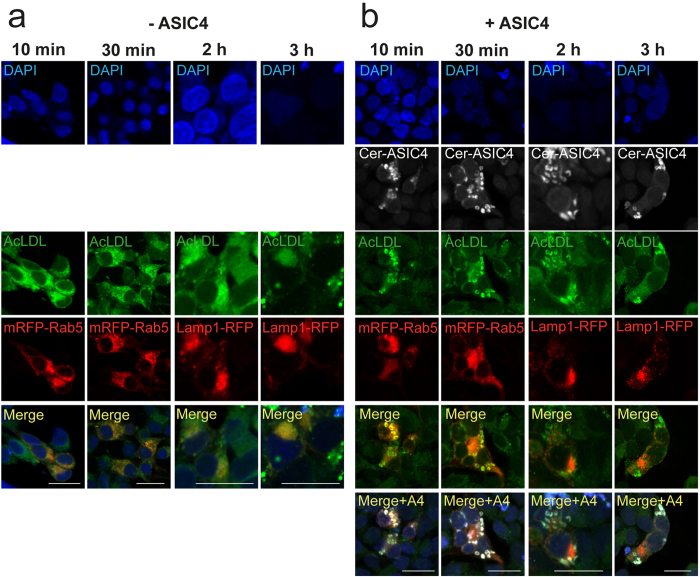
Endocytosed AlexaFluor488-labeled acetylated low-density lipoprotein (AcLDL) gets trapped in early endosomes in ASIC4-expressing HEK293 cells. (**a**) Alexa488-AcLDL co-localized with mRFP-Rab5 after 10 min and 30 min and with Lamp1-RFP after 2 h and 3 h of incubation at 37 °C in HEK293 cells. (**b**) In cells expressing Cerulean-ASIC4 (Cer-ASIC4), Alexa488-AcLDL co-localized with mRFP-Rab5 after 10 min and 30 min but not with Lamp1-RFP after 2 h and 3 h, showing that ASIC4 impaired the trafficking towards lysosomes. Images are representative of at least three independent experiments (n = 3–5). Merged images show an overlay of the DAPI (blue), Alexa488 (green) and mRFP (red) channels for the –ASIC4 condition. Due to some bleed-through of fluorescence emission from the Cerulean to the DAPI channel, for the +ASIC4 conditions merged images exclude DAPI and Cerulean fluorescence to better recognize the red/green overlap. The Merge +A4 panel shows the overlay of all four channels. Scale bars: 20 μm.

We wondered whether the large vacuolar-like structures that formed upon over-expression of ASIC4 are bona fide early endosomes or some specialized organelles that served to deposit ASIC4. To address this question we investigated the passage of low-density lipoprotein (LDL) along the endocytic pathway in cells over-expressing ASIC4. We incubated HEK293 cells, which were transiently transfected with mRFP-Rab5 or Lamp1-RFP, with acetylated LDL (AcLDL) labelled with AlexaFluor488. In cells not over-expressing ASIC4, AcLDL appeared in early endosomes after 10 and 30 min as revealed by overlap of fluorescence with mRFP-Rab5 in these cells (Fig. 4a). After 2 and 3 h, AcLDL had reached lysosomes as revealed by overlap of fluorescence with Lamp1-RFP. In contrast, in cells over-expressing Cerulean-ASIC4, AcLDL accumulated in the large early-endosome related vesicles, where they co-localized with ASIC4 (Fig. 4b), and even after 3 h there was no overlap of fluorescence with Lamp1-RFP (Fig. 4b). This result identifies ASIC4-expressing endosomes as bona fide endosomes of the endocytic pathway and suggests that over-expression of ASIC4 and formation of vesicles trapped endocytosed LDL in these early-endosome-related structures.

### The amino-terminus is important to direct ASIC4 into an early endosome-related organelle

**Figure 5:**
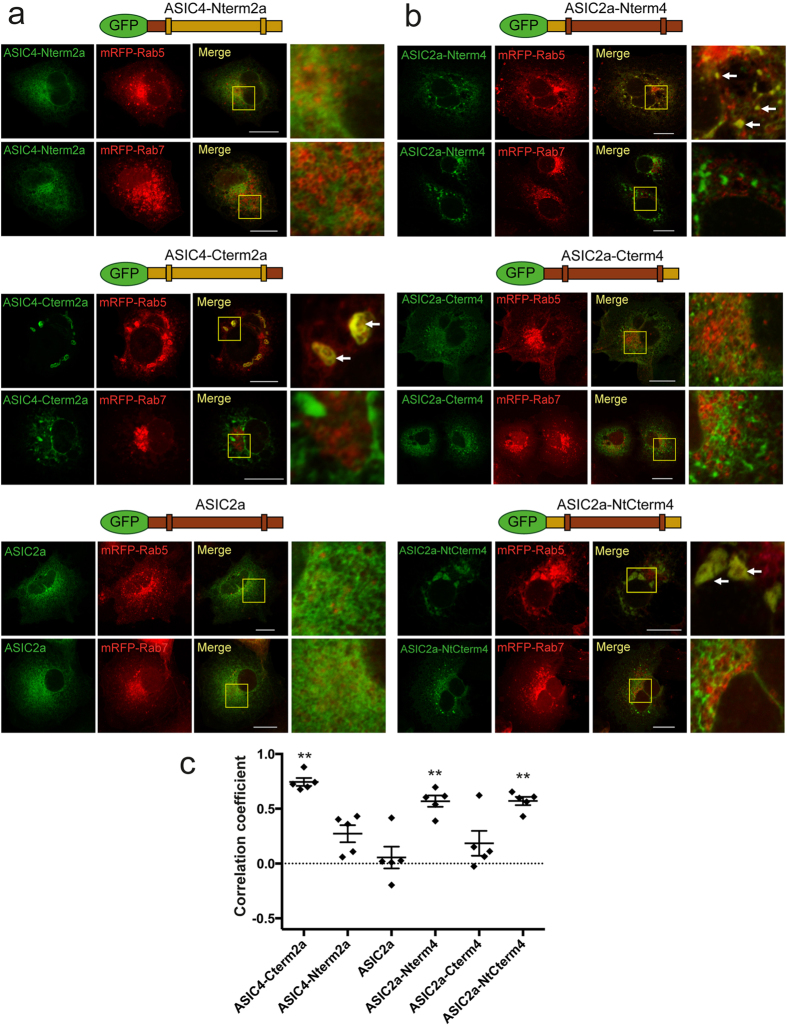
Localization of ASIC4 is determined by its cytoplasmic amino-terminus. (**a**) Chimeras, in which either the intracellular amino- or the intracellular carboxyl-terminus of ASIC4 was replaced by the corresponding parts of ASIC2a, were fused at their N-termini to GFP and co-expressed with either mRFP-Rab5 or mRFP-Rab7 in COS-7 cells. The chimera containing the amino-terminus of ASIC2a (ASIC4-Nterm2a) had a reticular distribution pattern whereas the chimera containing the carboxyl-terminus of ASIC2a (ASIC4-Cterm2a) had a vacuolar distribution and fluorescence overlapped with red fluorescence of mRFP-Rab5. Images on the right show magnifications of informative regions marked in the “Merge” images. Arrows indicate examples of fluorescence overlap. (**b**) A chimera of ASIC2a containing the amino-terminus of ASIC4 (ASIC2a-Nterm4) had a vesicular distribution and fluorescence overlapped with red fluorescence of mRFP-Rab5 (indicated by arrows) whereas a chimera of ASIC2a containing the carboxyl-terminus of ASIC4 (ASIC2a-Cterm4) had a reticular distribution pattern. A chimera of ASIC2a with both cytoplasmic termini of ASIC4 (ASIC2a-NtCterm4) localized in vacuolar-like structures like ASIC4. Scale bars: 20 μm. (**c**) Colocalization with mRFP-Rab5 was quantified by Pearson´s correlation coefficient. n = 5 representative images; one image of each condition is shown in (**a**) or (**b**) and the other four images in Supplementary Fig. S2 online. PCC values of chimeras were compared by Student´s t-test with that of ASIC2a and revealed a significantly higher co-localization with mRFP-Rab5 of the three chimeras containing the N-terminus of ASIC4 (ASIC4-Cterm2a, ASIC2a-Nterm4 and ASIC2a-NtCterm4); **p < 0.01.

Having identified early endosome-related vacuoles as the primary site of ASIC4 expression, we used the distinct distribution patterns of ASIC2a and ASIC4 to identify the parts of ASIC4 that are relevant for its location in these organelles. First, we constructed chimeras, in which we replaced either the intracellular amino-terminus of ASIC4 by the corresponding part of ASIC2a (chimera ASIC4-Nterm2a) or its intracellular carboxyl-terminus (chimera ASIC4-Cterm2a). The chimeras were fused to GFP. Similar to ASIC4, chimera ASIC4-Cterm2a localized in large vacuolar-like structures that co-stained with mRFP-Rab5 (PCC = 0.74 ± 0.04, n = 5; Fig. 5a,c and Supplementary Fig. S2 online). In contrast, chimera ASIC4-Nterm2a had a reticular distribution pattern and did not co-stain with mRFP-Rab5, similar to ASIC2a (PCC = 0.27 ± 0.08, n = 5; Fig. 5a,c and Supplementary Fig. S2 online), identifying the N-terminus of ASIC4 as necessary for its location in vacuolar structures.

We then did inverse chimeras. The chimera, in which we replaced the amino-terminus of ASIC2a by the amino-terminus of ASIC4 (chimera ASIC2a-Nterm4), localized to vesicular structures, which co-stained with mRFP-Rab5 (PCC = 0.57 ± 0.05, n = 5; Fig. 5b,c and Supplementary Fig. S2 online), indicating that the ASIC4 amino-terminus is sufficient to direct this chimera into early-endosome-related vesicles. The smaller size and number of these vesicles, however, indicates that this chimera did not fully reproduce the trafficking of ASIC4. In contrast, the chimera, in which we replaced the carboxyl-terminus of ASIC2a by the carboxyl-terminus of ASIC4 (ASIC2a-Cterm4), retained the reticular distribution of ASIC2a and showed no overlap of red and green fluorescence when co-expressed with mRFP-Rab5 (PCC = 0.18 ± 0.11, n = 5; Fig. 5b,c and Supplementary Fig. S2 online). Exchanging both termini together resulted in a chimera that localized in large vacuolar-like structures and co-localized with mRFP-Rab5 (PCC = 0.57 ± 0.04, n = 5; Fig. 5b,c and Supplementary Fig. S2 online). Together, these results identify the amino-terminus of ASIC4 as crucial for its location in early endosomal vesicles.

### The first eighteen amino acids of the N-terminus are important for the anterograde transport of ASIC4

**Figure 6:**
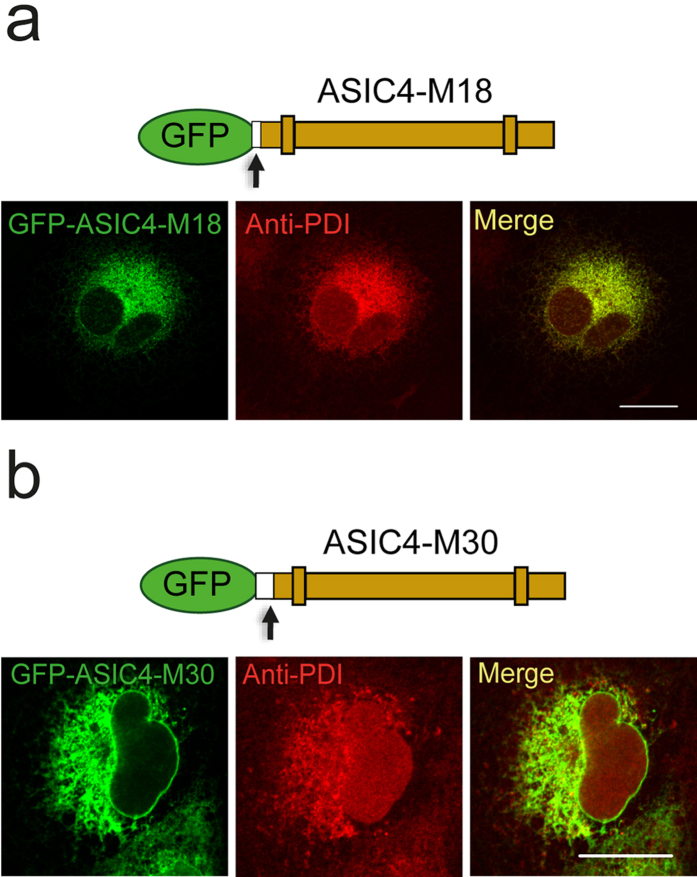
Amino-terminal truncations of ASIC4 retain the channel in the ER. (**a**) Truncation of the amino-terminus of ASIC4 at position 18 (GFP-ASIC4-M18) resulted in a reticular distribution pattern. (**b**) Truncation at position 30 (GFP-ASIC4-M30) had a similar effect. Antibody stainings revealed a co-localization of both truncated proteins with the ER-resident protein disulfide-isomerase (PDI) in COS-7 cells. Scale bars: 20 μm.

To confirm the role of the cytoplasmic amino-terminus for trafficking of ASIC4, we successively truncated it. When we removed the first eighteen amino-terminal amino acids (GFP-ASIC4-M18), the channel had a reticular distribution pattern (Fig. 6a), similar to ASIC2a. Stainings with anti-PDI antibody revealed co-localization, suggesting that GFP-ASIC4-M18 indeed localized to the ER (Fig. 6a). Thus, the first eighteen amino acids are important for the anterograde transport of ASIC4 and their truncation leads to a retention of the channel in the ER. A truncation of the first thirty amino acids (GFP-ASIC4-M30) including a di-leucine motif at position 29/30 (see below) resulted in the same reticular distribution pattern as truncation of the first eighteen amino acids (Fig. 6b). Co-stainings with the anti-PDI antibody confirmed a localization of GFP-ASIC4-M30 in the ER (Fig. 6b). These results confirm an important role of the amino-terminus of ASIC4 for its location in early endosome-related organelles.

### Mutation of a C-terminal di-arginine motif at position 478 changes the distribution pattern of ASIC4

**Figure 7:**
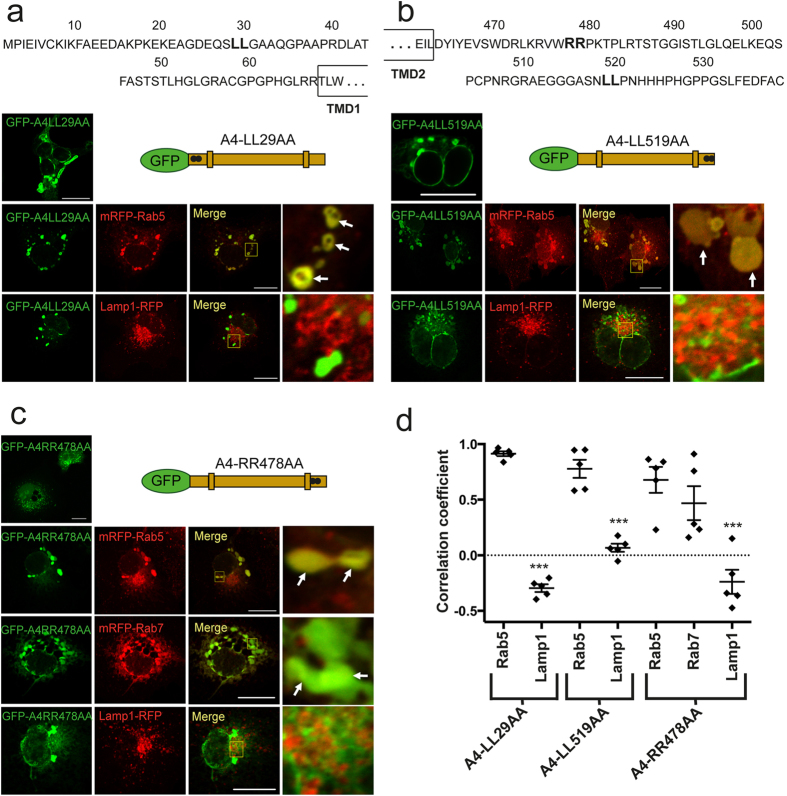
A cytoplasmic di-arginine motif is important for localization of ASIC4. (**a**) Top, amino acid sequence of the cytoplasmic amino-terminus of rat ASIC4; the di-leucine motif (L29L30) is highlighted in bold. Bottom, expression in COS-7 cells of ASIC4, in which the di-leucine motif had been mutated to alanines. To the left of the scheme, a representative image shows the vacuolar distribution pattern of the mutant (GFP-A4LL29AA). Below the scheme, co-expression with mRFP-Rab5 (early endosomes) or Lamp1-RFP (lysosomes) revealed clear overlap only with mRFP-Rab5. Images on the right show magnifications of informative regions marked in the “Merge” images. Arrows indicate examples of fluorescence overlap. (**b**) Top, amino acid sequence of the cytoplasmic carboxyl-terminus of rat ASIC4; the di-leucine motif (L519L520) and the di-arginine motif (R478R479) are highlighted in bold. Bottom, expression in COS-7 cells of ASIC4, in which the di-leucine motif at the carboxyl-terminus had been mutated to alanines. To the left of the scheme, a representative image shows the vacuolar distribution pattern of the mutant (GFP-A4LL519AA). Below the scheme, co-expression with mRFP-Rab5 (early endosomes) or Lamp1-RFP (lysosomes) revealed clear overlap only with mRFP-Rab5. (**c**) Expression in COS-7 cells of ASIC4, in which the di-arginine motif at the carboxyl-terminus had been mutated to alanines. To the left of the scheme, a representative image shows that the mutant (GFP-A4RR478AA) accumulated in smaller vesicles than ASIC4 wild type. Below the scheme, co-expression with mRFP-Rab5 (early endosomes) or with mRFP-Rab7 (late endosomes) showed clear overlap, while there was only slight overlap with Lamp1-RFP (lysosomes). Scale bars: 20 μm. (**d**) Colocalization of ASIC4 with mRFP-Rab5, mRFP-Rab7 and Lamp1-RFP was quantified by Pearson´s correlation coefficient. n = 5 representative images; one image of each condition is shown in (**a**–**c**) and the other four images in Supplementary Fig. S3 online. PCC values of the different marker proteins were compared with Rab5 by Student’s t-test and revealed a significantly higher co-localization with mRFP-Rab5 than Lamp1 for all three mutants. For the mutant of the di-arginine motif (A4-RR478AA), co-localization with mRFP-Rab7 was not significantly different from co-localization with Rab5; ***p < 0.001.

ASIC4 contains two di-leucine motifs at its cytoplasmic termini, which often mediate endocytosis of membrane proteins^34^, one at its amino-terminus (LL29/30; Fig. 7a) and one at its carboxyl-terminus (LL519/520; Fig. 7b). To investigate whether the di-leucine motifs of ASIC4 are relevant for its vacuolar distribution pattern, we substituted them by alanines (GFP-A4LL29AA and GFP-A4LL519AA, respectively) and assessed the effect of these substitutions on ASIC4 localization in COS-7 cells (Fig. 7a,b). Examination with confocal laser scanning microscopy revealed that cells expressing GFP-A4LL29AA or GFP-A4LL519AA showed an accumulation in vacuolar structures, similar to ASIC4 wild-type (Fig. 7a,b). Co-expression of the mutants with mRFP-Rab5 confirmed their accumulation in early endosome-related vacuoles (PCC = 0.91 ± 0.02 and 0.78 ± 0.08, respectively, n = 5; Fig. 7a,b,d and Supplementary Fig. S3 online). In contrast, they did not co-localize with Lamp1-RFP, the marker for lysosomes (PCC = −0.30 ± 0.03 and 0.07 ± 0.04, respectively, n = 5; Fig. 7a,b,d and Supplementary Fig. S3 online). We conclude that the two di-leucine motifs of ASIC4 are not required for its accumulation in early endosome-related vacuoles.

In addition to the di-leucine motifs, ASIC4 contains a di-arginine motif, another known trafficking signal^35^, at its carboxyl-terminus (RR478/479; Fig. 7b). We substituted both arginines by alanines (GFP-A4RR478AA) and assessed the effect of these substitutions on ASIC4 localization in COS-7 cells (Fig. 7c). Cells expressing GFP-A4RR478AA showed an accumulation of the channel in vesicles (Fig. 7c, top picture). While GFP-A4RR478AA did still co-localize with mRFP-Rab5 (PCC = 0.68 ± 0.12, n = 5; Fig. 7c,d and Supplementary Fig. S3 online), it also co-localized with mRFP-Rab7 (PCC = 0.47 ± 0.15, n = 5; Fig. 7c,d and Supplementary Fig. S3 online), suggesting that this mutant partially localized to late endosomes. We conclude that the di-arginine motif is important for efficiently retaining ASIC4 in early endosome-related vacuoles and for efficiently preventing its further transport along the endo-lysosomal pathway.

**Figure 8:**
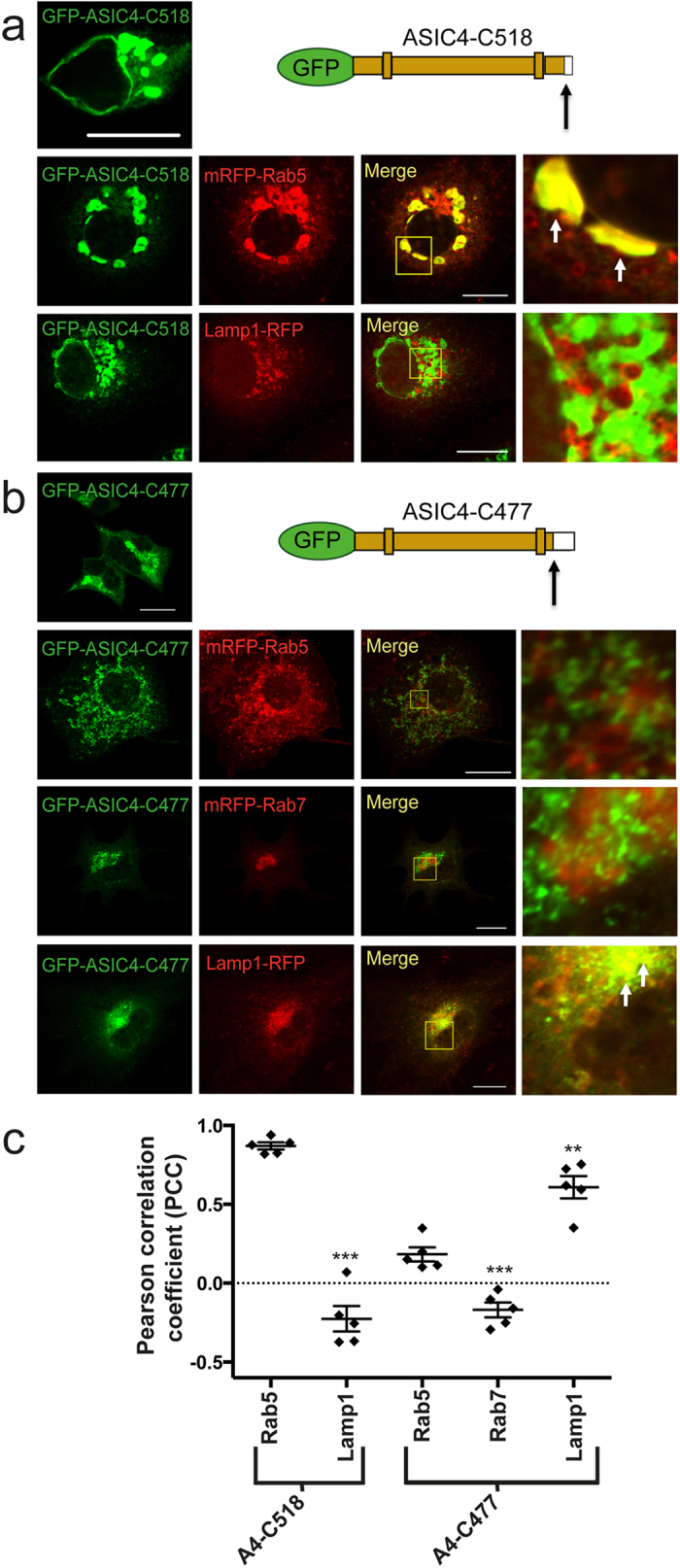
A large truncation of the carboxyl-terminus of ASIC4 directs the channel into lysosomes. (**a**) To the left of the scheme, a representative image illustrates that truncation of the carboxyl-terminus of GFP-ASIC4 at position 518 (GFP-ASIC4-C518), including the di-leucine motif at position 519,520, resulted in accumulation in large vesicles when transfected in COS-7 cells. Below the scheme, there was clear overlap with mRFP-Rab5 (early endosome marker) and no overlap with Lamp1-RFP (lysosome marker), respectively. Images on the right show magnifications of informative regions marked in the “Merge” images. Arrows indicate examples of fluorescence overlap. (**b**) To the left of the scheme, a representative image illustrates that truncation of the carboxyl-terminus of GFP-ASIC4 at position 477 (GFP-ASIC4-C477), including the di-arginine motif at position 478,479, resulted in predominant localization in small vesicles. Below the scheme, GFP-ASIC4-C477 overlapped with fluorescence from Lamp1-RFP (lysosomes) but not from mRFP-Rab5 (early endosomes) or mRFP-Rab7 (late endosomes). Scale bars: 20 μm. (**c**) Colocalization with mRFP-Rab5, mRFP-Rab7 and Lamp1-RFP was quantified by Pearson´s correlation coefficient. n = 5 representative images; one image of each condition is shown in (**a**,**b**) and the other four images in Supplementary Fig. S4 online. PCC values of the different marker proteins were compared with Rab5 by Student´s t-test and revealed a significantly higher co-localization with mRFP-Rab5 than Lamp1 for the truncation GFP-ASIC4-C518 but the opposite for truncation GFP-A4-C477; ** p < 0.01, *** p < 0.001.

We corroborated these findings by successively truncating the carboxyl-terminus of ASIC4. When we truncated the last twenty-one amino acids (GFP-ASIC4-C518), including the di-leucine motif at position 519/520 (Fig. 7b), cells expressing GFP-ASIC4-C518 showed a vacuolar distribution pattern and co-localized with Rab5 (PCC = 0.86 ± 0.02, n = 5; Fig. 8a and Supplementary Fig. S4 online), similar to ASIC4 wild type. This shows that the last 21 amino acids of the carboxyl-terminus, including the di-leucine motif (LL519/520), are dispensable for the accumulation of ASIC4 in early endosome-related vacuoles.

When we truncated the last 62 amino acids of the carboxyl-terminus of ASIC4 (GFP-ASIC4-C477) including the di-arginine motif at position 478/479 (Fig. 7b), however, we observed an accumulation of the truncated channel in small vesicles (Fig. 8b). The distribution pattern of GFP-ASIC4-C477 resembled the distribution pattern of GFP-A4RR478AA. To identify the vesicles to which GFP-ASIC4-C477 localized, we co-transfected the truncation with mRFP-Rab5, mRFP-Rab7 and Lamp1-RFP. In contrast to GFP-A4RR478AA, GFP-ASIC4-C477 co-localized with Lamp1-RFP (PCC = 0.65 ± 0.07, n = 5) but not with mRFP-Rab7 (PCC = −0.14 ± 0.05, n = 5); it also did not co-localize with mRFP-Rab5 (PCC = 0.18 ± 0.04, n = 5; Fig. 8b and Supplementary Fig. S4 online). These results suggest that GFP-ASIC4-C477 accumulated in lysosomes. Since the 62-amino acids truncation left only ~15 amino acids of the cytoplasmic carboxyl-terminus (Fig. 7b), it is possible that this truncation led to entry into a degradative pathway.

## Discussion

Our study shows that ASIC4 has a subcellular location, which is unique among heterologously expressed ASICs: it mainly resides in endosome-related vacuoles. Location of ASIC4 in large vacuolar organelles was striking but consistently observed, in two different cell systems, and with and without a GFP-tag. It is likely that vacuoles formed because of the over-expression of ASIC4. We considered that location in an early endosome-related organelle is a consequence of aggregation of over-expressed ASIC4. In this case, however, we would have expected that the vacuolar-like structures were related to the ER and not to early endosomes. Our assay testing endocytosis of LDL also identified ASIC4-expressing vacuoles being within the endo-lysosomal pathway. Furthermore, truncation of only 18 amino-terminal amino acids resulted in a reticular distribution pattern as for ASIC2a and mutation of the carboxyl-terminal di-arginine motif resulted in the appearance of ASIC4 in late endosomes. Both results are unexpected if ASIC4 formed aggregates in the ER. They rather suggest that ASIC4 specifically accumulates within early endosomes. In summary, we conclude that endosomal location of ASIC4 in COS-7 and HEK293 cells was not a simple artefact due to aggregation or over-expression.

The amino-terminus of ASIC4 had a pivotal role for forward-trafficking of ASIC4 from the ER into early-endosomes. It was not only necessary for location of ASIC4 in early-endosome related vacuoles (Figs 5 and 6)but was also sufficient to direct ASIC2a to early-endosomes (Fig. 5). In addition, it appears to be responsible for the formation of large vacuolar-like structures.

Cytoplasmic amino-termini of ASIC1, ASIC2a and ASIC3 are approximately 40 amino acids long; the amino-terminus of ASIC4, however, is 25 amino acids longer^8^ and this amino-terminal extension is highly conserved in orthologs from other species^36^. It is also well conserved in ASIC1b^5^, where deletion of the domain increases current amplitudes 5- to 10-fold in *Xenopus* oocytes^5^,^37^, suggesting that it impairs surface expression. In zebrafish, ASIC4 has two paralogs, zASIC4.1 and zASIC4.2. zASIC4.1 is a proton-gated ion channel, whereas zASIC4.2 is insensitive to protons^36^. Similar to ASIC1b, deletion of the amino-terminal domain of zASIC4.1 increases current amplitudes and surface expression about 10-fold^38^. The amino-terminal domain of ASIC4 contains several lysine residues and it has been proposed that ASIC4 gets polyubiquitinated at these lysines^13^, which may lead to enhanced degradation and reduced surface expression. Lysines are not present in the amino-terminal domain of ASIC1b, however, indicating that the presence of lysine residues and polyubiquitination cannot fully explain the role of this domain in surface expression of ASICs. Moreover, in our study we found no evidence for location of ASIC4 in lysosomes. Our results rather suggest that in ASIC4 the amino-terminal domain is important for forward trafficking into an early-endosome-related compartment. Although the exact role of this domain may be different in different ASICs, the previous observations that deletion of the domain dramatically increases surface expression of ASICs is consistent with an important role in trafficking.

In addition to the amino-terminal domain, we found evidence that a C-terminal di-arginine motif has a role in retaining ASIC4 in early-endosomes and preventing further trafficking into late endosomes/lysosomes (Fig. 7). The presence of chimeras ASIC4-Cterm2a and ASIC2a-Nterm4, which contain the ASIC2 C-terminus lacking the C-terminal di-arginine motif, in Rab5-positive vesicles suggests that this motif has a more subtle role in localizing ASIC4 to early endosome-related vesicles than the amino-terminal domain. Di-arginine motifs are known to retrieve some membrane proteins to the ER^35^. Retrieval is mediated by interaction with the coat protein complex I (COPI). Somewhat reminiscent of this situation, the di-arginine motif at position 478/479 of ASIC4 seems to retrieve the channel to early endosome-related vacuoles. Whether the di-arginine motif of ASIC4 interacts with COPI has to await further studies. Recently, a di-arginine motif in the proximal carboxyl-terminus of ASIC1a has been shown to interact with the adaptor protein complex and to mediate constitutive endocytosis of ASIC1a^39^.

Another feature of ASIC4 that fits with its location within the endo-lysosomal pathway is its high glycosylation, which is characteristic for proteins of acidic organelles like lysosomes. Most ASICs contain two to four N-glycans, whereas ASIC4 has eight consensus sequences for N-glycosylation most if not all of which are used^40^. Together, these results suggest that ASIC4 might routinely passage through or even reside in intracellular organelles that are related to early endosomes. Based on the expression of its RNA, ASIC4 has highest expression in pituitary gland^8^,^23^. At present we cannot exclude that ASIC4 localizes in its native cells in an unknown organelle not present in COS-7 or HEK293 cells and that early endosome-related vacuoles in heterologous cells are a surrogate of this organelle. In the future, it will therefore be important to identify the subcellular location of ASIC4 in cells that express it endogenously.

It should be emphasized that we cannot rule out that a small fraction of the total ASIC4 pool localizes to the plasma membrane. While mutation of two di-leucine motifs, which enhance endocytosis of other membrane proteins^34^, had no effect on the distribution of ASIC4, this is no strong evidence against plasma membrane expression of ASIC4.

An intracellular location would explain why the function of ASIC4 remains unknown. Whereas all other ASICs assemble into proton-activated channels and physiological functions are emerging for them^41^, activating stimuli and physiological functions of ASIC4 are unknown^8^,^9^. Previously, we also found no evidence that ASIC4 assembles into functional heteromeric ASICs^8^. Others, however, have reported that ASIC4 down regulates the expression of other ASICs^13^. Retention by ASIC4 of other ASIC subunits in an intracellular organelle could reconcile these findings. In the future it will therefore be important to investigate whether co-expression of ASIC4 with other ASICs changes their subcellular location.

In *Xenopus* oocytes, zASIC4 reaches the plasma membrane^38^. Since oocytes are a permissive expression system, we speculate that zASIC4 reaches the oocyte plasma membrane because their control systems are less stringent than that of HEK293 or COS-7 cells, used in the present study. In zASIC4-expressing oocytes, removal of extracellular divalent cations and low pH elicit a small sustained current, which is unselective for cations and insensitive to amiloride^38^. Thus, ASIC4 seems to mediate a current that is atypical for an ASIC. If ASIC4 had an intracellular location, current evidence, thus, suggests that it could carry a monovalent cation current. Cations possibly involved include Na^+^, K^+^ and protons. At present, we can only speculate on the function of this conductance, but studies with isolated ASIC4-expressing endosomes or studies investigating pH of such endosomes might give first hints.

Collectively, our results show that in heterologous expression systems ASIC4 localizes to endosome-related vacuoles. While many questions regarding its function and location in its native environment remain open, it should be considered that ASIC4 is a resident ion channel of an intracellular compartment.

## Methods

### Plasmids and antibodies

Chimeras and truncations of rat ASIC4 were constructed by recombinant PCR and point mutations by quick-change mutagenesis. All constructs were cloned into pcDNA3.1 (Invitrogen, Eugene, Oregon, USA), pEGFPC1, pEGFPC2 (Clontech) or pCerulean (Addgene). Other plasmids encoding organelle markers were received from Addgene. mRFP-Rab5 (Addgene plasmid 14437) and mRFP-Rab7 (Addgene plasmid 14436) were provided by Ari Helenius^31^, DsRed-Rab11 (Addgene plasmid 12679) was provided by Richard Pagano^33^, and Lamp1-RFP (Addgene plasmid 1817) was provided by Walther Mothes^32^.

Antibodies used were: anti-PDI (ab3672, Abcam, Cambridge, UK), anti-giantin (PRB-114C, Covance, Münster, Germany), anti-EEA1 (#2411, Cell Signaling, Danvers, MA, USA or #610456, BD Transduction Laboratories), Alexa Fluor® 488-conjugated anti-rabbit, Alexa Fluor® 633-conjugated anti-rabbit, and Alexa Fluor® 635-conjugated anti-mouse (Invitrogen). The polyclonal anti-ASIC4 antibody was raised against a synthetic peptide (HPHGPPGSLFEDFAC) corresponding to the C-terminus of rat ASIC4^8^. The peptide was coupled via glutaraldehyde to keyhole limpet hemocyanin and used to raise polyclonal antisera in rabbits (Charles River Laboratories, Kisslegg, Germany). The antiserum was purified by affinity chromatography with the synthetic peptide. For endocytosis assay, Alexa488-conjugated acetylated low density lipoprotein (Alexa Fluor® 488 AcLDL) was used (Invitrogen).

### Cell culture and transfections

Cell culture material including media and supplements were from Biochrom (Berlin, Germany). Cells were cultured in a humidified atmosphere with 5% CO_2_ at 37 °C. Monkey kidney (COS-7)and human embryonic kidney (HEK293) cells were cultured in Dulbecco’s modified Eagle medium (DMEM) supplemented with 10% fetal bovine serum (FBS), 5% L-glutamine and 1% pyruvate (Biochrom).

Heterologous cells were transiently transfected using X-treme gene 9 transfection reagent (Roche, Mannheim, Germany) or FuGene6 (Promega, Mannheim, Germany) according to manufacturers´ protocols. For antibody staining, transfection of single constructs and co-localization studies with organelle markers, cells were grown on 13 mm glass coverslips and transfected with 1 μg of each construct. For endocytosis assay, HEK293 cells were transfected with 2 μg of each construct. Cells were incubated for 24 h after transfection before staining, fixation with 4% paraformaldehyde, or endocytosis assays with Alexa Fluor® 488 AcLDL.

### Immunofluorescence

24 h after transfection, coverslips were washed three times with PBS and fixed with PBS containing 4% paraformaldehyde for at least 10 min at room temperature. Cells were either directly mounted in Mowiol or Fluoromount-G (Southern Biotech, Biozol Diagnostica, Eching, Germany) or further used for antibody staining. For this purpose, cells were washed twice with PBS and permeabilized by incubation with PBS containing 0.3% Triton-X-100 for 20 min at room temperature. Cells were washed three times with PBS and incubated with blocking solution containing 10% FBS for 1 h. Coverslips were transferred to a humidified chamber and incubated with the first antibody (1:200 dilution) in blocking solution overnight at 4 °C. Cells were washed three times with PBS and incubated with Alexa Fluor® 633-conjugated anti-rabbit or Alexa Fluor® 635-conjugated anti-mouse secondary antibody (1:500 dilution) for 1 h at 37 °C. After two washing steps, coverslips were dried and mounted in Mowiol.

For antibody stainings using anti-EEA1 or anti-ASIC4, cells were washed three times with PBS and permeabilized with PBS containing 2% normal goat serum (NGS; Pan Biotech, Aidenbach, Germany) and 0.1% Triton-X-100 for 10 min at room temperature (RT). Cells were incubated with the first antibody (1:100 dilution) in PBS containing 2% NGS for 1 h at RT. Afterwards, cells were washed three times with PBS, blocked in PBS containing 2% NGS for 10 min at RT and incubated with either Alexa Fluor® 488-conjugated anti-rabbit, Alexa Fluor® 633-conjugated anti-rabbit, or Alexa Fluor® 635-conjugated anti-mouse secondary antibody (1:500 dilution) for 1 h at RT. To stain the nuclei, cells were incubated with PBS containing DAPI (1:5000 dilution) for 5 min at RT prior to washing them three times with PBS and mounting them in Fluoromount-G.

Fluorescence imaging was performed with a LSM 700 confocal microscope (Carl Zeiss, Jena, Germany) with 40× oil-immersion objective, 4× line averaging, 200 mHz scanning speed and pinhole set to 1 Airy unit.

### Quantification of co-localization

Co-localization was quantified using Pearson´s correlation coefficient (PCC), which measures the pixel-by-pixel covariance in the signal levels of two images^42^. PCC values range from 1 to −1. Two images whose fluorescence intensities are perfectly, linearly related have a value of 1 and two images whose intensities are perfectly, but inversely, related have a value of −1. Probes that are uncorrelated with one another are expected to have a PCC value close to zero^42^.

PCC was determined with the Zen Blue Software 2012 (Zeiss). Background in the GFP- and RFP-channels were chosen manually for each image by using the pipette tool and PCC was then calculated automatically by the software. At least 5 different, representative images from 2–3 independent transfections were analysed. Bar diagrams in Figs 5, 7 and 8 represent results from five representative images for each condition; the corresponding images are shown in the figures and in the supplementary Figs 2–4. Data are expressed as means ± SEM. Student′s t-test was used to compare experimental groups; p values <0.05 were considered as statistically significant. Images, for which PCC was not determined, are representative for images of at least three independent transfections.

### Endocytosis assay

For internalization studies, Alexa Fluor® 488 AcLDL (5 μg/ml) was added to transiently transfected HEK293 cells, which were seeded on coverslips, 24 h post-transfection and was allowed to internalize for 10, 30 (for mRFP-Rab5 transfectants), 120, or 180 min (for Lamp1-RFP transfectants) at 37 °C. HEK293 cells were either transfected with mRFP-Rab5, Lamp1-RFP, mRFP-Rab5 and Cerulean-ASIC4, or Lamp1-RFP and Cerulean-ASIC4. At the end of incubation, cells were put immediately on ice, and washed three times with ice-cold PBS. Afterwards, they were fixed with 4% paraformaldehyde for 30 min at RT, washed again with ice-cold PBS and mounted in Fluoromount-G.

## Additional Information

**How to cite this article**: Schwartz, V. *et al.* Acid-sensing ion channel (ASIC) 4 predominantly localizes to an early endosome-related organelle upon heterologous expression. *Sci. Rep.* **5**, 18242; doi: 10.1038/srep18242 (2015).

## Supplementary Material

Supplementary Information
