# Supplementary material for: Acid-sensing ion channel (ASIC) 4 predominantly localizes to an early endosome-related organelle upon heterologous expression
Source: Sci Rep. 2015 Dec 15;5:18242. doi: 10.1038/srep18242 (PMC4678866; doi:10.1038/srep18242)
Supplement: Supplementary Information [file srep18242-s1.pdf]

Acid-sensing ion channel (ASIC) 4 predominantly localizes to an early endosome-related organelle upon heterologous expression

Verena Schwartz, Katharina Friedrich, Georg Polleichtner[¶], and Stefan Gründer*

Institute of Physiology, RWTH Aachen University, Pauwelsstrasse 30, D-52074 Aachen, Germany

Supplementary Figures

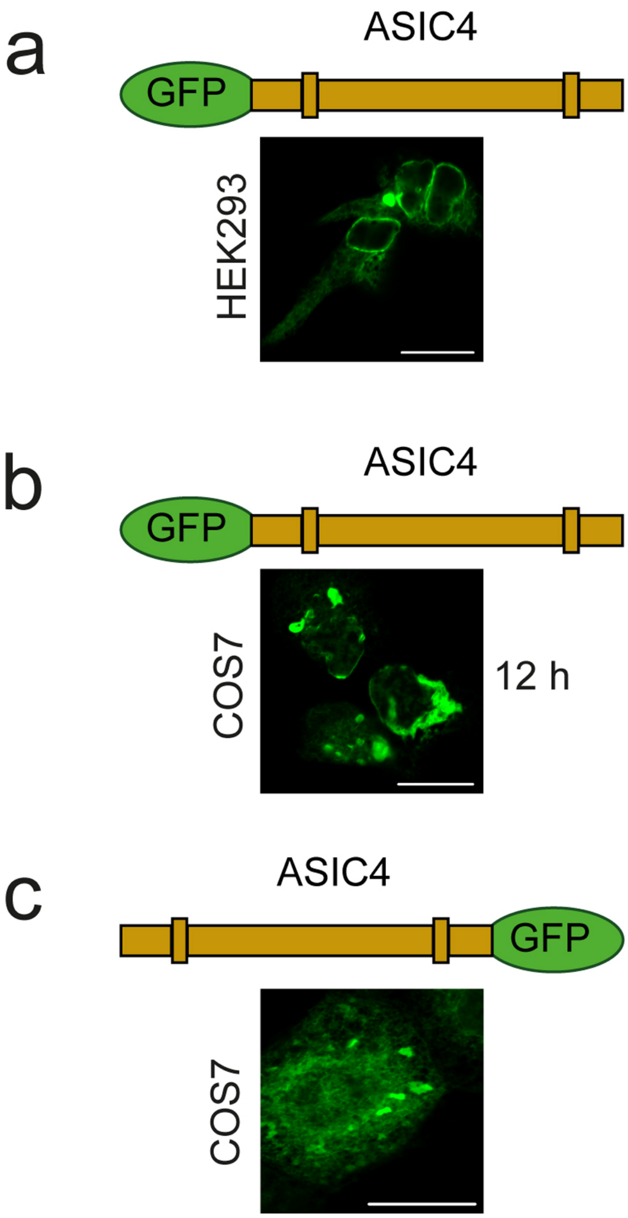

Fig. S1: Vacuolar expression pattern of ASIC4 is independent of the heterologous cell system, duration of expression and position of the GFP-tag. (a) 24 h expression of amino-terminally GFP-tagged ASIC4 (GFP-ASIC4) in HEK293 cells. (b) 12 h expression of GFP-ASIC4 in COS-7 cells. (c) 24 h expression of carboxyl-terminally GFP-tagged ASIC4 (ASIC4-GFP) in COS-7 cells.

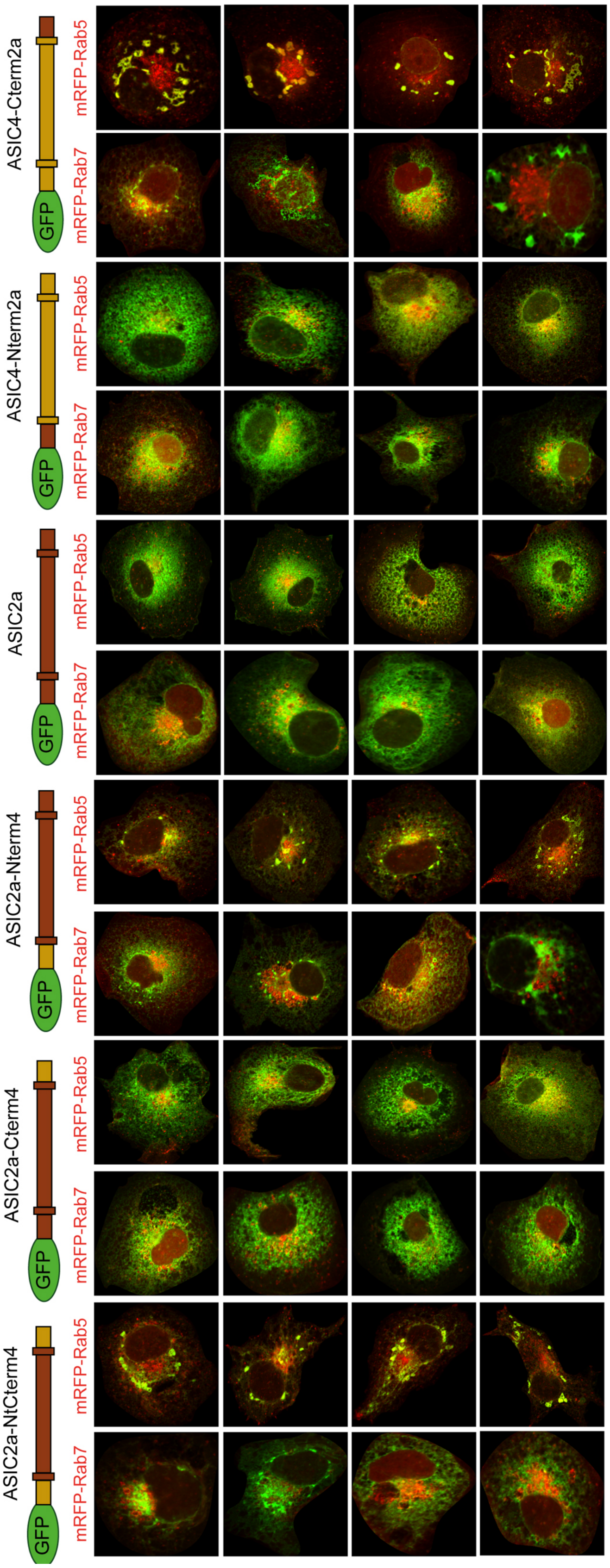

Fig. S2: Localization of ASIC4 is determined by its cytoplasmic amino-terminus. Four additional images to Figure 5. PCC values in the bar diagram of Figure 5c derive from these four images plus the one image shown in Figure 5.

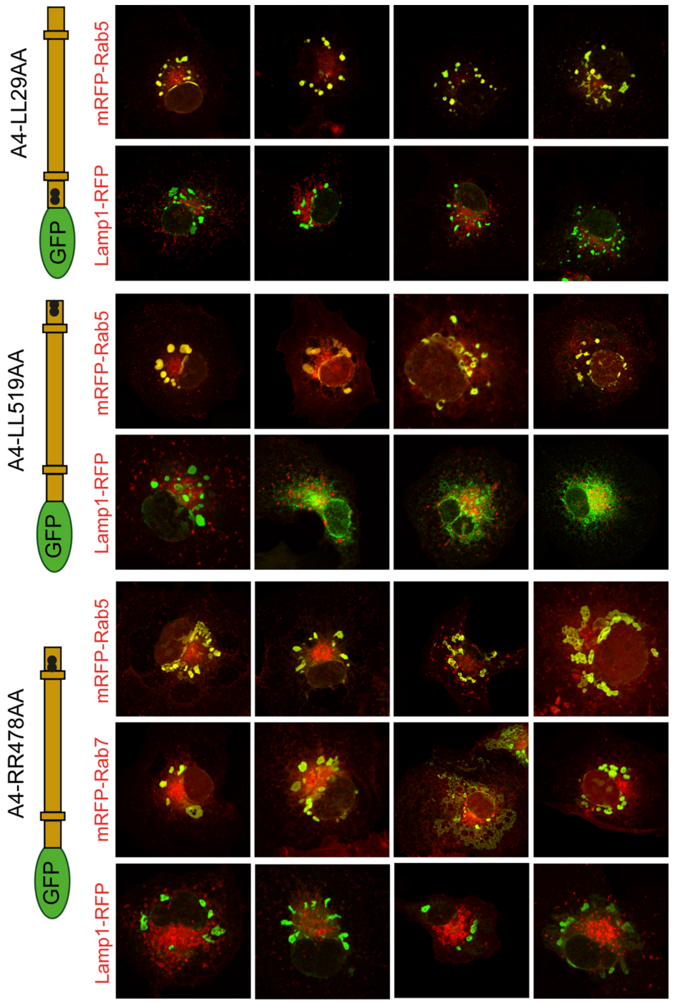

Fig. S3: A cytoplasmic di-arginine motif is important for localization of ASIC4. Four additional images to Figure 7. PCC values in the bar diagram of Figure 7d derive from these four images plus the one image shown in Figure 7.

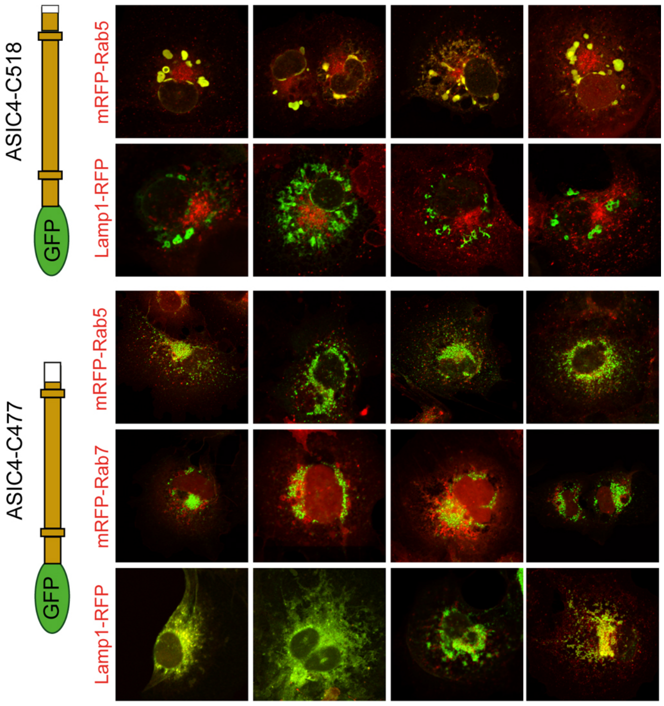

Fig. S4: A large truncation of the carboxyl-terminus of ASIC4 directs the channel into lysosomes. Four additional images to Figure 8. PCC values in the bar diagram of Figure 8c derive from these four images plus the one image shown in Figure 8.
